# The two languages of science

**DOI:** 10.1186/s13059-020-02057-5

**Published:** 2020-06-17

**Authors:** Itai Yanai, Martin Lercher

**Affiliations:** 1 Institute for Computational Medicine, NYU Langone Health, New York, NY 10016 USA; 2 Institute for Computer Science & Department of Biology, Heinrich Heine University, 40225 Düsseldorf, Germany

“If we allow ourselves the license of talking about genes as if they had conscious aims, always reassuring ourselves that we could translate our sloppy language back into respectable terms if we wanted to, we can ask the question, what is a single selfish gene trying to do?”—Richard Dawkins, *The Selfish Gene*: p. 88

“I’ve made agents out of system 1 and system 2 because everybody finds it easier to think about agents – with propensities and traits – than about abstract categories. Agents make powerful subjects because they’re active and they do things.”—Daniel Kahneman, personal communication

In no small part, the amazing success of modern science in learning about our world is rooted in its rigorous structure: scientists clearly lay out assumptions, design conclusive experiments with the appropriate controls, and use statistical methods to assess our confidence in the results. These processes form the core of what Francois Jacob called *day science* [1, 2]. To utilize these strengths, we speak in a highly precise and metaphor-free language when we describe our work in manuscripts and at conferences. Acquiring this language is an important part of our formal training as day scientists. But there is an equally important second language of science, which is rarely taught explicitly and often overlooked. This is the language of *night science*—the creative part of our work, where we come up with the first, hazy versions of the very ideas to be tested by day science [1, 2]. In night science, insistence on precision becomes a hindrance, while metaphors and anthropomorphizing—the personification of non-human objects such as cells, proteins, and genes—impart us with powerful intuitions about the unknowns we explore. Once we have identified an idea in our night science explorations, we can translate it into respectable day science language, so that an experiment can be designed and executed using the rigorous day science tools at our disposal. To avoid misunderstandings, the distinct language of night science must be clearly labeled when we speak. But developing it as a complement to the formal day science language is an important step in our development as a creative scientist.

## Do not anthropomorphize genes, they do not like it

“We are survival machines – robot vehicles blindly programmed to preserve the selfish molecules known as genes” [3]. When it was first written down in the preface to Richard Dawkins’ *The Selfish Gene*, this sentence was shocking. Could anyone seriously suggest that genes have human features, such as selfishness, or the ability to program something or someone? This sort of anthropomorphism—the attribution of human qualities and intentions to non-human entities—was not meant to be taken literally. Dawkins was well aware that genes cannot actually be selfish; to think so would be ridiculous. But his anthropomorphic language created a powerful, compelling image, visualizing the central role of genes during evolution.

Human speech is *chock-full* of metaphors: it has been estimated that in trying to *capture the imagination* of our *brothers in arms*, we *deploy* several metaphors a minute [4]. (There were four in the previous sentence.) Anthropomorphisms are a special type of metaphor that is particularly prevalent in informal speech among scientists. At the same time, many scientists consider them to be inappropriate in scientific discourse, arguing that metaphors and anthropomorphisms belong to the realm of poetry, while science is the domain of rigor and precision. Calling a gene “selfish,” we are taught, can lead to miscommunication and misunderstandings. Indeed, it has been argued that anthropomorphic images of inter-microbial warfare in microbiology have misdirected research and prevented scientists from understanding the diverse functions of “anti-bacterial” small molecule secretion [5]. As a consequence, students are taught not to anthropomorphize when thinking or talking about biology [6], and many a student has had their assignment edited in red ink by their professor with the epitaph “Do not anthropomorphize!”

The case against anthropomorphism in science is simple and straightforward: genes, proteins, and cells do not “feel” or “want” anything, they are not driven by intentions, but instead move and change based on physical and chemical forces. Anthropomorphic thinking is typical of reasoning in early childhood, and understanding the true underlying causes of inanimate “behavior” is an important step in maturation [7]. So why are anthropomorphisms still ubiquitous in informal scientific thoughts and discussions [8]? They are not restricted to biology but are found across all sciences; anthropomorphisms are particularly widespread in artificial intelligence research, where they have been characterized as “at best misleading and at worst downright dangerous” [9]. Is anthropomorphic language a bad habit that we should all strive to eradicate, or could a case be made for its usefulness?

## The intentional instinct

A defining feature of the human animal is its reliance on social structures and interactions. Throughout our evolution, a high level of social cognition was crucial: on the one hand, our livelihoods frequently depended on collaborations within a population; on the other hand, much of prehistoric human mortality is believed to have been caused by other humans [10]. Even today, to cooperate and occasionally compete with other people, we must understand and predict their actions with reasonable speed and accuracy. Our brains achieve this by taking an “intentional stance,” utilizing a “theory of mind”: we infer beliefs, desires, and intentions, and we then use them to predict corresponding actions [11, 12]. It is thus reasonable to suggest that the human brain has evolved under selective pressures that favored fast, intuitive predictions for the behavior of other humans based on their presumed intentions.

This idea is backed up by neurological research. Much of our body’s energy budget is consumed by the brain, and most of our brain’s energy is spent on spontaneous activity that continues when the brain is apparently at rest [13]. The brain regions showing most of this costly activity coincide with areas associated with adopting an intentional stance [14–18]. Functional MRI studies have even suggested that spontaneous activity of these regions primes the brain to adopt an intentional stance when presented with a problem [17].

**Fig. 1:**
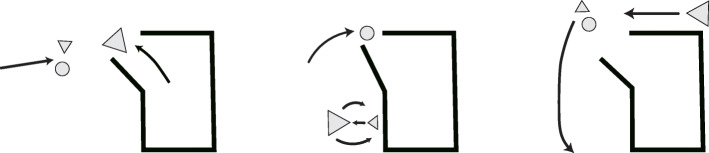
The Heider-Simmel animation. An animated movie involving two triangles and a circle leads us to tell a story filled with motivations and purpose. Redrawn from [19]

Thus, our brains may have been optimized by evolution for processing information that is cast in an intention-based framework. This specialization is analogous to that of GPUs, which are optimized for processing image-based information—but which can be repurposed for any problem that can be cast in a mathematically equivalent form. As an example of how we intuitively take an intentional stance when we process neutral information, consider the Heider and Simmel animation involving two triangles and a circle moving around (Fig. 1) [19]. When people are asked to describe what they saw in this animation, many tell a story about the motivations and emotions of each of these objects. Our impulse as humans, it seems, is to tell stories with metaphorical language and anthropomorphisms—as inappropriate as this may seem from the perspective of scientists doing science. Indeed, experiments in psychology have shown that humans solve problems faster when primed to think of a metaphor or analogy for the task at hand [20].

## The two languages

It is obvious why science values precision: the ability to rigorously test ideas is a triumph over illogical thinking. While one can claim anything regarding nature—that water has memory, that the location of the stars predicts our personal fortunes, and that an apple a day keeps the doctor away—science has the means to test these hypotheses and throw out (i.e., falsify) the ones that fail. And to make sure that a test is logically valid, we must express the tested hypothesis in precise, metaphor-free language.

The day science language required to describe the testing of hypotheses is the one used almost exclusively in scientific manuscripts, in which we start with a hypothesis, rationalize it (often in retrospect), and then proceed to test it. The language of day science is spoken at conferences, where we attempt to convince our colleagues of our findings. In grant proposals, day science language takes center stage as we describe an approach for rigorously testing an important hypothesis. It is also the exclusive language of journal clubs and manuscript reviews, where the testing of a scientific claim forces us to rigorously challenge the underlying hypothesis, to see if it can withstand the challenge.

While the language of day science facilitates the precise formulation and testing of scientific ideas, it is not particularly conducive to the creation of new ideas. As humans, we require a language permissible to intuition—one that gives us a “feeling” for the phenomenon. This is the language of night science. In night science language, we are allowed to anthropomorphize freely, helping us to grasp why and how something may be happening. Night science language is not precise, but what we lose in rigor, we gain in intuition. We ask “what does the organism want?” We put ourselves into the shoes of a gene. We ponder the best strategy for a genome in a given situation.

Night science language appears to commit the error of attributing desires and intentions to non-human entities such as genes, proteins, and cells. But while this kind of language seems inaccurate and misleading, in many ways it is simply shorthand for respectable day science language. Scientific statements expressed in the anthropomorphizing terms of night science can be rephrased in the language of day science. Examples are given in Table 1. One of these is taken from our book *The Society of Genes*, where we wrote that “a cancer gene aims to secure an unfair advantage” [25]. Of course a gene does not try to do anything—a gene just is. What we meant can be rephrased more rigorously as “a mutation to a proto-oncogene that causes an increased growth rate of the cells that carry the mutation will over time lead to an increase in the total fraction of body cells that carry the mutation.” When we express this concept in night science language, we know that we can translate it back to rigorous day science language. Yet we gain an intuition for the physiological and evolutionary processes the cancer gene is involved in by thinking of it as though it had intentions, and this helps us to think faster and deeper about tumorigenesis. Night science language exploits a particular bias of our brain circuitry, drawing upon its well-honed techniques for revealing intentions and basing projections on them.

**Table 1:** Translating night science language to respectable day science language

Night science	Day science
“Nature *abhors* a vacuum” (attributed to Aristotle)	“Effusion or movement towards lower pressure occurs because unobstructed gas molecules will become more evenly distributed between high- and low-pressure zones, by a flow from the former to the latter.” [21]
“A much more demanding *task* for these enzymes is to *discriminate* between similar amino acids ... However, the observed error frequency in vivo is only 1 in 3000, indicating that there must be subsequent *editing* steps to enhance fidelity. In fact the synthetase *corrects* its own errors ... How does the synthetase *avoid* hydrolyzing isoleucine-AMP, the *desired* intermediate?” [22] as cited by [23]	“Each aminoacyl-tRNA synthetase is highly specific for a given amino acid. Indeed, a synthetase will incorporate the incorrect amino acid only once in 10^4^ or 10^5^ catalytic reactions. How is this level of specificity achieved?” [24], in a later edition of the same textbook.
“A cancer gene *aims* to secure an *unfair* advantage.” [25]	“Mutations to a proto-oncogene that cause an increased growth rate of the cells that carry the mutation will over time lead to an increase in the total fraction of body cells that carry the mutation” [25]
“We are *survival machines – robot vehicles blindly programmed* to preserve the *selfish* molecules known as genes.” [3]	“Genes are the sole replicators in biological evolution. [...] As fascinating as all the complex adaptations that have arisen through selection may be, the results of this process matter in selection only if they are reflected in the content of their respective replicators.” [26].
“The image of a relatively *smooth [fitness] landscape*, where populations adapt by *going up-hill* once they fix an advantageous mutation, are *trapped* in mountain peaks and remain isolated from other possibly higher fitness maxima by deep valleys, often appears as the way in which adaptation proceeds.” [27]	Evolutionary adaptations of a population can be quantified by fitness changes due to the fixation of mutations that increase fitness. Such increases may lead to genotypes with locally maximal fitness, i.e., fitness cannot increase further through additional point mutations, as any individual such mutation would first lead to a strong decrease in fitness.
“Non-hazardous bacteria also *help* prevent diseases by occupying places that the pathogenic, or disease-causing, bacteria *want* to attach to. Some bacteria protect us from disease by *attacking* the pathogens.” [28]	Commensal bacteria with no direct detrimental effects on human health often benefit humans by occupying ecological niches in the human body that could alternatively be occupied by disease-causing bacteria, thereby reducing their potential fitness. Some bacteria release compounds toxic to pathogens, thereby reducing the probability of disease for their host.

The typical questions you may be asking yourself or your colleagues differ substantially between the two languages of day and night science (Table 2). To apply the intentional stance in biology, harnessing the intuition founded in our brains’ ability to predict intention-based behavior, you should ask questions that would sound silly in day science. If you were the protein, what would be your goal? If you were the cell, what would you try to achieve with a certain regulatory pattern? This language not only lacks rigor, but may even be misleading; of course, genes, proteins, and cells are not rational agents with beliefs and desires. But this is the nature of night science: anything goes, as long as it may potentially give you valuable ideas about a system. The ideas may turn out to be completely wrong when subjected to the tests of day science, but along the way, we may figure out something important about the underlying system. For the lay public in particular, night science language allows for an intuition about a scientific topic; this is why anthropomorphisms and other metaphors abound in the popular science literature. Without a science apprenticeship, such intuition by itself does not easily lead to a translation to precise day science language, but the understanding it conveys is powerful.

**Table 2:** Distinct questions in the two languages of science

Night science questions	Day science questions
What does that protein want?	Is it necessary and sufficient?
Why would the cell do something that stupid?	What is the significance (*P* value)?
How does the cell know what to do?	What is the mechanism?
Why did the cell not know that it has been invaded by the virus?	Is there a negative control and a positive control?
How do these cells know to stop dividing?	Is the proposed experiment sufficiently powered?

Of course, the notion of there being two languages in science—a night science one and a day science one—is itself an analogy: both use the same words and grammar. But they are as distinct in terms of modes of thinking as perhaps the liberal arts and sciences were when C. P. Snow complained that they were “two cultures” [29]. There is good reason to keep the two languages separate, emphasizing the distinction between the generation of ideas and their subsequent testing. This distinction is the hallmark of modern science. In contrast, alchemists typically did not distinguish between day and night science. They believed in a deeper, fundamental truth behind metaphors, blurring the distinction between metaphorical and real relationships [30]. Without a clear distinction between ideas and tests, their science was severely hampered.

## The language of discovery

**Fig. 2:**
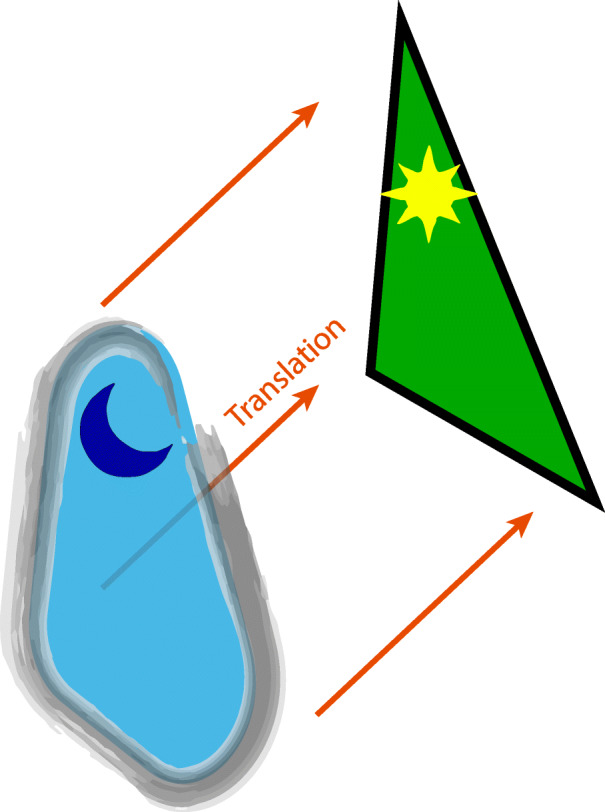
The two languages of sciences. Night science ideas—which may be hazy and incompletely formed—are initially expressed with anthropomorphisms and other metaphors, but can be translated eventually into the precise language of day science

Night science language not only helps to provide an intuition about complex ideas. For the generation of some important new ideas, it is absolutely necessary, as not every metaphorical night science idea is translatable one-to-one from the outset into precise day science language in the way shown in Table 1. A truly new idea is born only roughly formed—there may not even be words for it yet. To make that idea falsifiable and thus testable by day science, it needs to be reshaped and refined, and an inexact yet intuitive language is all we initially have for that task (Fig. 2) [31].

As an example, consider selfish genes and how the use of this anthropomorphic language enabled the discovery of ultra-selfish elements in the genome. Since the organism is only a “survival machine,” a gene may “choose” to be so selfish that it does not provide any useful contribution to organismal fitness at all. This notion has led to the discovery of selfish DNA: genes that can secure their survival in the genome with a copying mechanism that provides a survival strategy without an additional function for the organism [32, 33]. Translated into day science, this idea has now been well established with the recognition and characterization of transposable elements. Of course, these may also acquire functions beneficial to the fitness of the entire genome, but this should not distract from their origin as purely selfish elements.

As another example of how night science language is conducive to discovery, we consider the history of a study we published recently [34]. Bo Xia used single-cell RNA-Seq to generate a gene expression dataset of cells during spermatogenesis. We found that thousands of genes are expressed after meiosis, when the genome is haploid. During one discussion, one of us remembered the observation that any given gene is likely to be expressed in the testes, for reasons that were not well understood. We noted that it was *crazy* for the germ cells to *turn on* so many genes at this vulnerable state, because the process of transcription could irreparably damage the DNA that is *intended* to form the genetic basis of the next generation. This led us to think that perhaps the cells *want* more mutations, as genetic variation is the engine of evolutionary adaptation. We decided to cross-reference our dataset with the known DNA variations in the human population to test this provocative idea. Popping back into day science, Bo performed the analysis and found the exact opposite of the expected result—genes expressed during spermatogenesis have not more, but in fact fewer mutations than those that are unexpressed. We then realized that the cells may instead *want* fewer mutations in these genes. We found evidence that the mechanism of transcription-coupled repair could be at play and reasoned that the widespread gene expression in the male testes may function exclusively to correct mutations by a process we termed “transcriptional scanning.” While the anthropomorphizing language of cells wanting to generate or correct mutations was inexact, it gave us an intuition for the system, leading us to the eventual discovery.

In a second recent article, Hugo Dourado developed a theoretical framework for the analysis of an optimal cellular *economy* [35]. While the eventual mathematical form of this theory is pure day science, its development was driven by questions about what bacterial cells *want*—they *want* to grow fast (as growth rate is related to fitness), and we reasoned that they *achieve* this by somehow *adjusting* the concentrations of their constituents. Again, we had to move back into day science to show that this intuition made sense, by solving the corresponding equations and comparing our predictions to the observed growth rate dependence of protein concentrations.

## Lost in translation

*The Selfish Gene* may be one of the most misunderstood books of all times, in no small part because of its anthropomorphic title. In public events, Dawkins has repeatedly answered questions about how stretches of DNA could be called selfish by pointing out that the questioner apparently did not read the footnote to the book’s title—the book itself. In the foreword to the book’s 30th anniversary edition, Dawkins went so far as to claim that he regrets the anthropomorphism in its title—in retrospect, he should have taken a friend’s advice and called the book “The Immortal Gene.” But Dawkins does not really mean it. *The Selfish Gene* provided its readers with an intuition for how to think about genes as the central target of natural selection, and hence as the agents of the story of life on this planet.

So why do anthropomorphisms cause so much aggravation? A lot of it can be resolved if we clearly distinguish between the realms of night science and day science and their respective languages. As has been pointed out over and over again, confusion and misunderstandings can arise when we bring anthropomorphisms into the world of day science. If we decide to venture into night science language during a scientific talk or in a journal article, we are leaving the default day science mode. To not confuse our audience, we should explicitly note that we are venturing into night science territory, but that everything we are about to say can be translated into rigorous day science language—if that is indeed the case.

Irritation can also result inversely, when an insistence on day science language infiltrates a night science discussion. When our brain operates in its creative mode, we should not be expected to speak precisely about an idea only being born; this is not the time for day science aspects such as statistics and controls. For example, when speaking in night science language about how a cancer cell might want to evade the immune system with a specific strategy, hearing your interlocutor reply “You know, cells do not actually want anything” may bring your flow of thoughts to a halt.

Our scientific training is conducted in the language of day science: in its terms, we learn how to design an experiment with negative and positive controls, how to find hidden assumptions, and how to use appropriate statistics. This pattern may lead to the assumption that it is the only language appropriate for science. Yet it is equally important to learn the language of night science, in which we try to see the world from the vantage point of your object of study—a gene, a protein, a cell—and ask “What does it want?” Night science language has its place wherever we want to develop an intuition, by taking advantage of the way our brains are wired to adopt an intentional stance, predicting what an object will do given its inferred beliefs and intentions [12]. In many ways, thinking and talking in night science mode may be more related to what Daniel Kahneman has called “fast thinking,” which draws on instinctive heuristics and is intuitive and emotional; conscious, rigorous day science may be closer to the deliberate and logical “slow thinking” mode [36]. We need both of those modes, and scientists advising their students to not anthropomorphize (instead of telling them *when* to anthropomorphize) may be doing science a disfavor. Night science language unlocks the potential for fast, intuitive exploration; in it we all become poets, and for the better of science.
